# Preparation of Two-Dimensional Polyaniline Sheets with High Crystallinity via Surfactant Interface Self-Assembly and Their Encryption Application

**DOI:** 10.3390/polym16091285

**Published:** 2024-05-03

**Authors:** Zhiwei Li

**Affiliations:** 1 Key Laboratory for Polymeric Composite and Functional Materials of Ministry of Education, School of Chemistry, Sun Yat-sen University, Guangzhou 510275, China; lizhw36@mail2.sysu.edu.cn; 2 State Key Laboratory of Optoelectronic Materials and Technologies, Sun Yat-sen University, Guangzhou 510275, China

**Keywords:** two-dimensional, crystallinity, interface self-assembling, polyaniline, encryption

## Abstract

In recent years in the field of traditional materials, traditional polyaniline has faced a number of scientific problems such as an irregular morphology, high difficulty in crystallization, and difficulty in forming an ordered structure compared to the corresponding inorganic materials. In response to these urgent issues, this study determines how to prepare a highly ordered structure in polyaniline formed at the gas-liquid interface. By dynamically arranging aniline monomers into a highly ordered structure with sodium dodecyl benzene sulfonate (SDBS) surfactant, aniline polymerization is initiated at the gas-liquid interface, resulting in two-dimensional polyaniline crystal sheets with a highly ordered structure. By elucidating the microstructure, crystallization process, and molecular structure of the two-dimensional polyaniline crystal sheets, the practical application of polyaniline as an encryption label in the field of electrochromism has been further expanded, thus making polyaniline widely used in the field of information encryption. Therefore, the synthesis of flaky polyaniline crystal sheets has a role in scientific research and practical application, which will arouse the interest and exploration of researchers.

## 1. Introduction

Two-dimensional polymers consist of a highly organized covalent (with covalent bond strength) network structure with a single atom or a molecular thickness that can be infinitely extended and self-supporting [1,2,3]. The highly organized structure affords two-dimensional polymers with unique physical and chemical properties. Thus, they have broad application prospects in fields such as optoelectronic devices, sensors, catalysis, and energy conversion and storage [4,5,6]. There are two types of two-dimensional polymers: inorganic two-dimensional polymers and organic two-dimensional polymers. Inorganic two-dimensional polymers mainly include graphene, boron nitride, and graphyne [7,8,9]. Generally, inorganic two-dimensional polymers are formed in nature or obtained through high-energy experimental processes such as chemical vapor deposition, high-temperature cracking, and molecular epitaxial growth [10,11,12]. These harsh experimental environmental conditions make it difficult to easily regulate the structure, properties, and functions of inorganic two-dimensional polymers at the molecular scale. Correspondingly, organic two-dimensional polymers can be prepared using chemical methods under mild laboratory conditions, and they can also be obtained through the construction of reasonable precursors [13,14,15,16]. For example, two-dimensional polymers can be designed and synthesized using monomers of different sizes and morphologies as well as containing specific functional groups, π−π conjugation, electron donors, or electron acceptors [17,18,19,20]. The research on organic two-dimensional polymers has mainly focused on material synthesis, structural characterization, and application functions. The specific progress of these researchers in the field of two-dimensional polymers includes the following: (1) By controlling dynamic covalent or chelating reactions at the liquid-liquid interface, multi-layer crystal films with lateral dimensions up to micrometers or even centimeters could be obtained [21,22]. The prepared materials have certain application value in field-effect transistors, photoelectric conversion, electrochromism, and electrocatalytic hydrogen production [23,24,25]. (2) A three-dimensional layered covalent organic framework material, such as polycrystalline powder, was obtained through a dynamic covalent chemistry method and then peeled off to obtain a two-dimensional polymer multilayer film with lateral dimensions up to micrometers [26,27]. (3) Borate ester chemical experimental methods were used to polymerize and form two-dimensional polymers [28]. (4) Rigid symmetric monomers were polymerized through dynamic imine chemistry at the gas-liquid interface to form a single-layer crystal film, and the application of the single-layer film in field-effect transistors was explored [29]. These studies indicate that research on organic two-dimensional polymers is still in its early stages and that there are still many issues that need to be addressed [17,30,31,32]. A large amount of applied basic research is needed on the molecular design, required synthesis methods, process control, reaction mechanisms, and relationship between structure and performance in organic two-dimensional polymers.

In order to solve the above difficulties, this study proposes a delicate research approach: for the synthesis of two-dimensional polyaniline crystals with regular shapes, a highly ordered inducer film was formed at the gas-liquid interface to explore the intrinsic interaction mechanism of surfactant molecules (SDBS) and aniline monomers dynamically self-assembled and arranged into a highly ordered structure, triggering polymerization reactions at the gas-liquid interface of aqueous solutions. Moreover, the aniline monomer polymerization reaction without SDBS surfactant induction was systematically investigated. By studying the effects of the SDBS surfactant molecules on the formation of organic two-dimensional polyaniline crystals through interfacial polymerization under different experimental conditions, it was found that the SDBS surfactant played an extremely important role in the gas-liquid interface self-assembly reaction that resulted in the formation of the ordered polyaniline crystal structure [33,34,35]. In view of this, this study aimed to prepare a method for controlling the synthesis and properties of two-dimensional polyaniline crystal sheets, especially in synthesizing uniform, hexagonal regular two-dimensional polyaniline crystal sheets at the gas-liquid interface. Based on the interpretation of their microstructure, a controllable method for synthesizing two-dimensional sheet polyaniline crystals was proposed. In addition, the obtained two-dimensional flaky polyaniline crystal was used as an electrochromic label material for information encryption, and the conductivity of the prepared self-assembly two-dimensional flaky polyaniline crystal film was also characterized, further revealing the inherent relationship between the structure and the performance of polyaniline for further explorations.

## 2. Materials and Methods

### 2.1. Materials

Aniline (>99%), sodium dodecyl benzene sulfonate (SDBS), and ammonium persulfate (APS, (NH_4_)_2_S_2_O_8_) were obtained from Shanghai Macklin Biochemical Co., Ltd. (Building 1, No. 68 Huatuo Road, Pudong New Area, Shanghai, China). Chloroform (CHCl_3_) and hydrochloric acid (HCl, 36–38%) were purchased from Guangzhou Baijun Technology Co., Ltd. (No.10 Shixin Road, Dalong Street, Panyu District, Guangzhou, China). All the chemicals were used as received.

### 2.2. Synthesis of Flaky Polyaniline (F-PANI) and Amorphous Polyaniline (A-PANI)

Flaky polyaniline (F-PANI) was synthesized in a 100 mL petri dish (diameter ~6 cm), and 50 mL of deionized (DI) water was poured into the dish. Then, 20 μL of SDBS solution (1 mg mL^−1^ in chloroform) was spread onto the water surface using a pipette gun and kept for an hour to allow for the chloroform to evaporate, after which a monolayer of SDBS formed at the gas-liquid interface of the water. Subsequently, 1 mL of aniline solution (10 μL, aniline) and 1 mL of HCl solution (pH = 1.0) were injected into the above solution. After 2 h, 2 mL of ammonium persulfate (APS, 2 mg) solution was injected into the liquid phase at a slow rate. After the injection, the crystallizing dish was covered with a glass cover. The above reaction was carried out at 0 °C for self-assembly polymerization, and then the reaction was maintained for 2 weeks. Then, F-PANI could be transferred onto copper substrates for further characterization (directly clamp the substrate with a polytetrafluoroethylene tweezer and then slowly scoop up the F-PANI film). The preparation of amorphous polyaniline (A-PANI) was similar to that of F-PANI but without the addition of the SDBS solution.

### 2.3. Characterization

An optical microscope (Olympus BX53M, Tokyo, Japan) was indirectly used to validate the quality and morphology of the crystallinity of the flaky polyaniline (F-PANI) and amorphous polyaniline (A-PANI). Scanning electron microscopy (SEM, Hitachi 4800, Tokyo, Japan) was performed to analyze the morphology of F-PANI and A-PANI. Transmission electron microscopy (TEM) and high-resolution transmission electron microscopy (HR-TEM) images were observed on a transmission microscope (FEI Themis Z, 300 keV, Thermo Fisher Scientific, Waltham, MA, USA) equipped with a spherical aberration corrector. GIWAXS (grazing-incidence wide-angle X-ray scattering) measurements were performed on a Xeuss 2.0 Small-Angle X-ray Scatterer (Xenocs, Grenoble, France) with an X-ray wavelength of 0.134144 nm and a MetalJet D2 liquid metal target light source. Raman spectra were measured on a microscopic confocal Laser Raman Spectrometer using a 532 nm laser at room temperature (Renishaw inVia, New Mills, UK). An atomic force microscope (AFM, dimension fastscan bio in peak force tapping mode in air) from Bruker was used. Fourier transform infrared (FT-IR) spectra of the samples were measured with a spectrometer (PerkinElmer Frontier, Waltham, MA, USA) with an attenuated total reflection (ATR) mode. The conductivity of the F-PANI film and A-PAN film was measured using a Jandel Cylindrical Probe combined with an RM3000 Test Unit (Jandel Engineering, Leighton Buzzard, UK). Cyclic voltammetry (CV) tests of the samples were performed on an electrochemical workstation (CHI660C, CH Instruments, Inc., Shanghai, China) at 0.05 V s^−1^ in a voltage range of −0.5 to 1.5 V.

## 3. Results and Discussion

### 3.1. Synthesis and Morphology of F-PANI and A-PANI

**Scheme 1:**
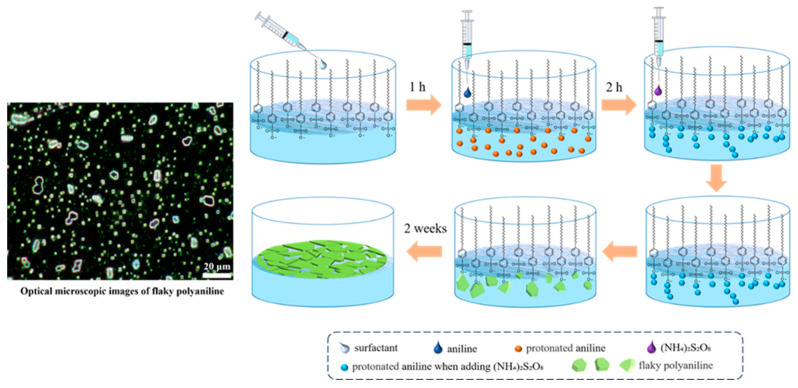
Schematic diagram of the F-PANI synthesis procedure.

By utilizing the SDBS surfactant at the gas-liquid interface, aniline monomers were effectively arranged on the surfactant through an interaction mechanism, forming a highly ordered structure in a dynamical and controllable manner. At the same time, the APS catalyst was used for kinetic catalysis, which triggered the corresponding polymerization of aniline monomers at the gas-liquid interface, resulting in the formation of flaky polyaniline (F-PANI). The preparation of amorphous polyaniline (A-PANI) was similar to that of F-PANI but without the addition of any surfactant solution. A schematic diagram of this process is shown in Scheme 1. At an ice bath temperature, a single-layer of SDBS surfactant was dispersed at the gas-liquid interface of the aqueous solution to form an ordered arrangement. Then, a certain amount of protonated aniline monomer aqueous solution was injected and left to stand for two hours without disturbance. It was found that, under the induction of surfactants, the protonated aniline monomers were self-assembled into an ordered structure. Subsequently, the prepared APS catalyst solution was slowly added to the surface of the above solution along the dish wall, and the two-dimensional sheet-like polyaniline crystal grew over two weeks. In the optical microscope image (Figure S1), it can be seen that some parts of the formed F-PANI have obvious edges and some parts do not. At the same time, the prepared A-PANI has no clear crystal particles. Moreover, under polarized light conditions at 90 degrees, the F-PANI crystal exhibited a bright yellow color, and the uniformity of the polyaniline crystal was well demonstrated on copper mesh (Figure S2). Under high magnification polarizing conditions, the F-PANI crystal appeared to have a flaky shape, which corresponds with the data obtained from SEM and TEM.

**Figure 1:**
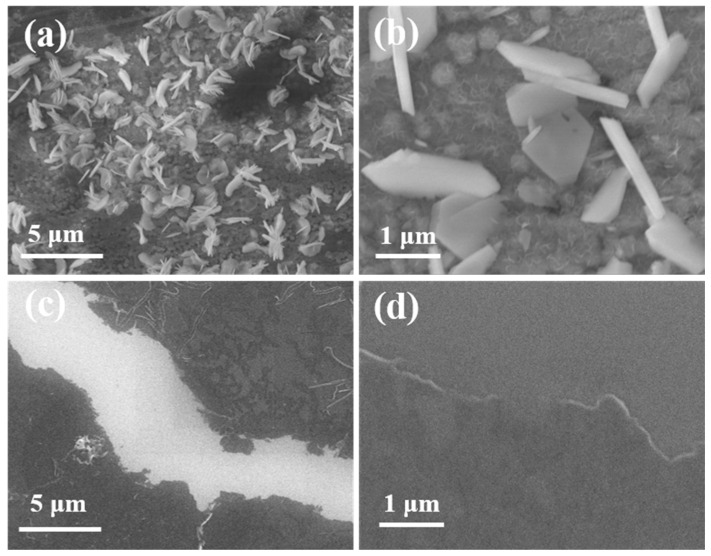
SEM images of F-PANI (**a**,**b**) and A-PANI (**c**,**d**).

F-PANI and A-PANI were characterized using SEM images, as shown in Figure 1. The low-magnification scanning electron microscopy images of the F-PANI and A-PANI clearly demonstrate different morphologies, as shown in Figure 1a,c. The morphology of the F-PANI presents as flakes that are densely adsorbed on the surface of the surfactant film, both horizontally and vertically. The A-PANI, however, has an amorphous morphology. The high-magnification electron microscope images in Figure 1b,d further demonstrated this distinction. The F-PANI mostly exhibits a regular morphology, including hexagonal, pentagonal, and quadrilateral shapes, with a thickness of approximately 250 nanometers. Some incomplete morphologies of the F-PANI were caused by external interference during the reaction, which interrupted the process of crystal growth.

Subsequently, to determine the thickness of the flakes of the F-PANI and A-PANI, we further characterized the samples using an force microscope (AFM). As shown in Figure S3a,b,e, the AFM image of the F-PANI displays a fragmentary hexagon with a thickness of ~250 nm, resulting from the crystal growth of the F-PANI being severely disrupted. Conversely, the AFM image of the A-PANI presents a disorganized morphology with uneven thickness and the thickness of the A-PANI film is about 50 nm, as shown in Figure S1c,d,f. The obtained AFM results are in good agreement with the SEM and optical microscopy images.

### 3.2. Structural Characterization of F-PANI and A-PANI

**Figure 2:**
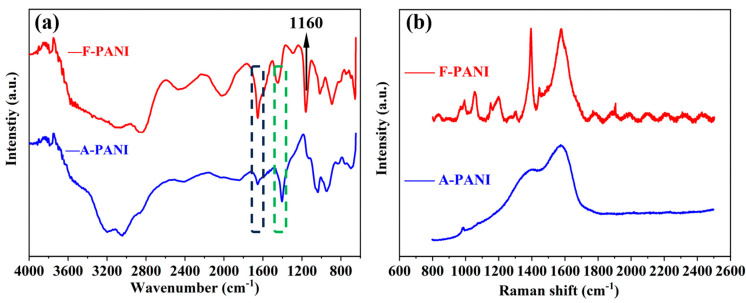
(**a**) FTIR spectra of F-PANI and A-PANI. (**b**) Raman spectra of F-PANI and A-PANI.

The infrared characteristic absorption spectrum of the F-PANI and A-PANI displayed in Figure 2a were examined to explore their crystallinity. The black dashed box indicates the characteristic absorption peak of the quinone ring structure, and the green dashed box indicates the characteristic absorption peak of the benzene ring structure [36,37,38,39]. Compared with the A-PANI, the peak in the F-PANI spectrum located at 1160 cm^−1^ was caused by the C-N stretching vibration, which is the characteristic absorption of protonated polyaniline crystals and the high degree of polymerization reaction. The Raman spectra in Figure 2b were used for further characterization of the structure of the F-PANI and A-PANI. The peak in the F-PANI spectrum at the positions of 1393 and 1575 cm^−1^ were interpreted as in-plane C-N stretching and out-of-plane-C=C-N bending, which indicate that the structure of the F-PANI contains excellent polymer crystals compared with that of the A-PANI [40,41,42,43].

**Figure 3:**
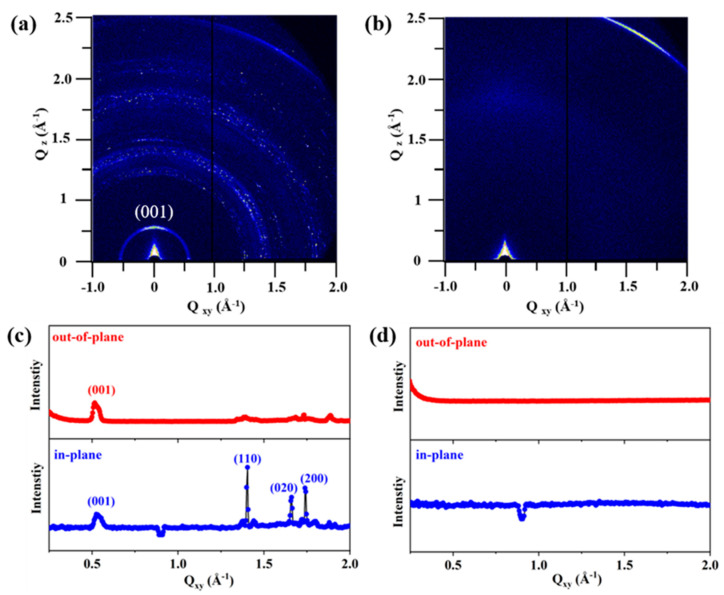
GIWAXS characterization of F-PANI film (**a**) and A-PANI film (**b**) on silicon. GIWAXS data in-plane and out-of-plane projections of F-PANI (**c**) and A-PANI (**d**).

To study the structure and crystallinity of the F-PANI and A-PANI more accurately, as shown in Figure 3, the GWAXS results also revealed the crystallization characteristics of the F-PANI and the amorphous characteristics of the A-PANI corresponding to the SEM images. The reflections in Figure 3a appear as rings because of the random orientation of the grains in the F-PANI sample in contrast to the substrate. As shown in Figure 3c, the out-of-plane diffraction signal of F-PANI corresponding to the (001) plane with a 12.1 Å spacing represents the stacking distance perpendicular to the substrate. In detail, the in-plane reflection rings of F-PANI at Qxy = 0.52, 1.40, 1.65 and 1.74 Å^−1^ correspond to the (001), (110), (020) and (200) lattice planes. Dure to the symmetry being forbidden, the (100) plane and (010) plane could not be shown in Figure 3a,c. The absence of (100) and (010) reflections further verified the p2gg plane group symmetry [44]. Furthermore, these results indicate that F-PANI is a monoclinic unit cell with a = 7.2 Å (100), b = 7.6 Å (010), c = 12.1 Å (001), and α = 97°, β = γ = 90° with the space group p2gg. In comparison, Figure 3b,d fully demonstrate that the out-of-plane and in-plane diffraction of A-PANI have no signals, confirming the absence of crystals.

**Figure 4:**
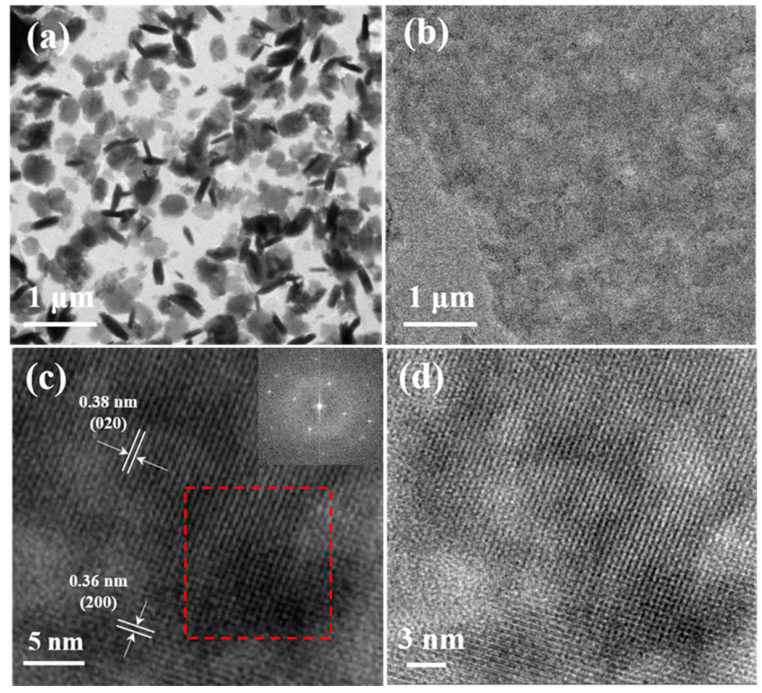
TEM images of F-PANI (**a**) and A-PANI (**b**). HRTEM image of F-PANI (**c**). Inset: The fast Fourier transform (FFT) image of the area selected by the red square. The inverse FFT image of the clear F-PANI lattice stripe (**d**).

**Figure 5:**
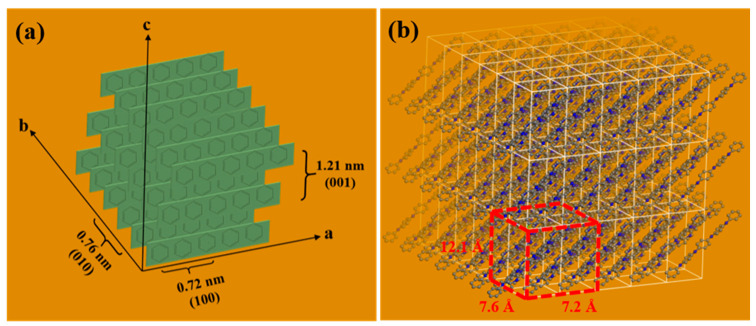
(**a**) The simplified crystal structure model of F-PANI. (**b**) The proposed simulated atomic model of F-PANI.

The low-magnification transmission electron microscope (TEM) image in Figure 4a,b is used to analyze the microstructure of the F-PANI and A-PANI. It is known that the morphology of the synthesized F-PANI is two-dimensional sheet with regular edges and incomplete zigzag edges. Among these sheets, some are stacked together and some are blending shapes. The rod-shaped (dark in color) and oval-shaped (light in color) are owing to the same F-PANI material. The arrangement and orientation of the prepared F-PANI are random, which some sheets standing up and some sheets lying flat. This phenomenon occurred when we irradiated with the TEM beam. Differently, the A-PANI film has no distinguishing features of crystallinity. The TEM characterization confirms the SEM characterization of the F-PANI and A-PANI. In order to further refine the crystal structure of the F-PANI, low-dose high-resolution TEM (HRTEM) is used to elucidate the atomic scale of the structural information. According to the literature reports, the low-dose TEM strategy to reduce radiation damage has been proven to be an effective method for achieving the atomic resolution of beam-sensitive samples [44,45,46]. The HRTEM image of the F-PANI obviously displays defined lattice stripes, which indicates the very high crystallinity of the F-PANI, as shown in Figure 4c. The inset image is the fast Fourier transform (FFT) of the area indicated by the red square, in which the bright dots form an approximate hexagonal shape, confirming the morphology identified via SEM and TEM. Moreover, the distinct lattice spacing of the F-PANI shows the crystal face (200) and (020), corresponding to ∼0.36 and 0.38 nm, respectively, which is in agreement with the GIWAXS result. In other words, the crystal face (100) and (010) correspond to ~0.72 and 0.76 nm. In order to clearly see the lattice stripes, the inverse FFT image of the F-PANI is shown in Figure 4d, which shows that it manifests excellent crystallinity. From the comprehensive analysis of the GIWAXS characterization and HRTEM image of the F-PANI, it can be seen that the polycrystalline diffraction ring at 1.65 Å^−1^ (020) should be corresponding to the repeating units along the chain, which indicates the typical layer-by-layer stacking structure like that of other 2D organic materials [47,48]. The simplified crystal structure model and the proposed simulated atomic model of the F-PANI are shown in Figure 5a,b, presenting a unit with cell parameters of 7.2, 7.6, and 12.1 Å.

### 3.3. Electrochromic Properties and Anti-Counterfeiting Label

To test the electrochromic performance of the F-PANI film and A-PANI film, we first tested the conductivity of the sample film of the F-PANI and A-PANI in Figure S4a,b. The F-PANI film and A-PANI film were deposited on a commercialized Au/Si substrate, and the entire process was carried out using the four-point probe method with the Jandel cylindrical probe combined with the RM3000 test unit. The electrical conductivity (σ) of the F-PANI film and A-PANI film were determined using the equation σ = 1/ρ = 1/R∗d, where R (Ω sq^−1^) is the sheet resistance calculated via the numerical value of I/V. The thickness (d) of the F-PANI film and A-PANI film were determined using the AFM with tapping mode, as shown in Figure S3. The resulting data indicate that the electrical conductivity of the F-PANI film and A-PANI film reached 133 S cm^−1^ and 95 S cm^−1^, respectively, due to the discontinuous and various sizes of the polyaniline crystal sheets loaded onto the polymer film, which result in a significant decrease in conductivity [44].

**Figure 6:**
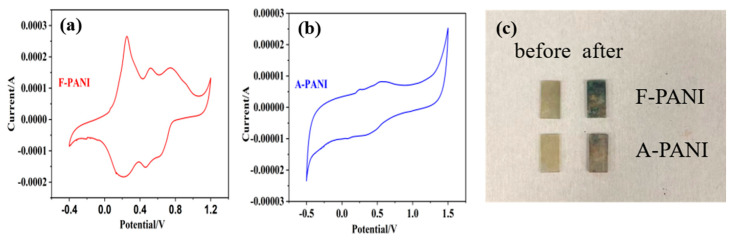
Electrochemical properties of F-PANI film. Cyclic voltammetry (CV) curves of F-PANI film (**a**) and A-PANI film (**b**) in aqueous solution of 2 mol/L H_2_SO_4_. (**c**) Photographic images of F-PANI and A-PANI film on the ITO as an anti-counterfeiting label before and after electrochromism.

We made further improvement to investigate the different electrochromic properties of the F-PANI film and A-PANI film as a potential anti-counterfeiting label. Cyclic voltammetry (CV) was used to study the electrochemical properties of the above polymer films [49]. The anti-counterfeiting labels consisted of the F-PANI film and A-PANI film deposited onto ITO glass, which was then assembled into a three-electrode system in 2 mol/L of H_2_SO_4_ (Figure 6). The cyclic voltammetric characteristics of the polymer films were tested using a CHI660C workstation equipped with a three-electrode system. The sample film/ITO conductive glass was used as the working electrode, a Ag/AgCl electrode was used as the reference electrode, a platinum plate was used as the auxiliary electrode, and 2 mol/L H_2_SO_4_ was used as the ion conductor. CV curves of the F-PANI film and A-PANI film were obtained with an applied voltage between −0.5 V and 1.5 V at a scanning rate of 0.05 V/s. By examining the CV curves of F-PANI and A-PANI, it was found that, in the forward scan, the polyaniline underwent a color transition from light to dark and then to light. In order to more clearly compare the colors exhibited by the polyaniline film, we selected the color images of each end potential cutoff, as shown in Figure 6c. Clearly, the F-PANI film, as an anti-counterfeiting label material, which utilizes the principle of electrochromism in practical applications, had a better color transition than the A-PANI film. The reason for this is mainly due to the presence of a large number of crystals in F-PANI film, which can accelerate the passage of charge carriers through the film loaded onto ITO [50].

## 4. Conclusions

This study aimed to establish a highly ordered material at the gas-liquid interface of aqueous solution through the interaction mechanism of SDBS surfactant, thereby dynamically arranging aniline monomers into a highly ordered structure. Then, at the gas-liquid interface, by controlling the binding of the SDBS surfactant with an inducer with aniline monomers accompanied by a specific catalyst, the polymerization of the aniline monomers was initiated, resulting in the formation of two-dimensional sheet-like polyaniline crystals. A new two-dimensional polyaniline crystal structure and molecular structure were obtained, thus establishing a method of preparing sheet-like two-dimensional polyaniline crystals. By elucidating the microstructure of the obtained two-dimensional polyaniline, it was revealed that the structure of polyaniline was closely related to the self-assembly of the SDBS surfactant at the gas-liquid interface. The relationship between the microstructure of the polyaniline and electrochromism was further explored, and a potential information encryption label was prepared. Ultimately, we are hopeful that this research on gas-liquid interface self-assembly can provide a new type of organic crystal two-dimensional material with a controllable structure that can be used for information encryption.

## Data Availability

Data are contained within the article.

## Supplementary Materials

The following supporting information can be downloaded at: https://www.mdpi.com/article/10.3390/polym16091285/s1, Figure S1: Optical microscopy images of F-PANI (a) and A-PANI (b), Figure S2: (a–d) Optical microscopy images of F-AN on the copper mesh, Figure S3: AFM images of F-PANI (a,b) and A-PANI (c,d). The height profiles of the image of F-PANI and A-PANI (e,f), Figure S4: The numerical value of I/V of the F-PANI film (a) and A-PANI film (b).
