# Supplementary material for: Preparation of Two-Dimensional Polyaniline Sheets with High Crystallinity via Surfactant Interface Self-Assembly and Their Encryption Application
Source: Polymers (Basel). 2024 May 3;16(9):1285. doi: 10.3390/polym16091285 (PMC11085674; doi:10.3390/polym16091285)
Supplement: Supplementary file 1 [file polymers-16-01285-s001.zip › polymers-2988900-supplementary.pdf]

polymers

MDPI

Article

Preparation of Two-dimensional Polyaniline Sheets with High Crystalline by Surfactant Interface Self-assembling and Their Encryption Application

Zhiwei Li [1,2]

[1] Key Laboratory for Polymeric Composite and Functional Materials of Ministry of Education, School of Chemistry, Sun Yat-sen University, Guangzhou 510275, China; lizhw36@mail2.sysu.edu.cn

[2] State Key Laboratory of Optoelectronic Materials and Technologies, Sun Yat-sen University, Guangzhou 510275, China

Abstract: In recent years in the field of traditional materials, traditional polyaniline has faced a number of scientific problems such as an irregular morphology, high difficulty in crystallization, and difficulty in forming an ordered structure compared to the corresponding inorganic materials. In response to these urgent issues, this study determines how to prepare a highly ordered structure in polyaniline formed at the gas−liquid interface. By dynamically arranging aniline monomers into a highly ordered structure with sodium dodecyl benzene sulfonate (SDBS) surfactant, aniline polymerization is initiated at the gas−liquid interface, resulting in two−dimensional polyaniline crystal sheets with a highly ordered structure. By elucidating the microstructure, crystallization process, and molecular structure of the two−dimensional polyaniline crystal sheets, the practical application of polyaniline as an encryption label in the field of electrochromism has been further expanded, thus making polyaniline widely used in the field of information encryption. Therefore, the synthesis of flaky polyaniline crystal sheets has a role in the scientific research and practical application, which will arouse the interest and exploration of researchers.

Keywords: Two−dimensional; crystalline; interface self−assembling; polyaniline; encryption

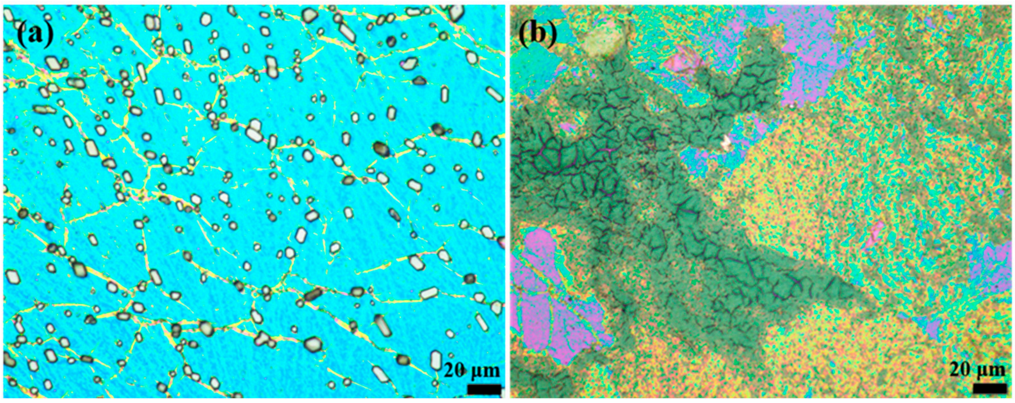

Figure S1. Optical microscopy images of F−PANI (a) and A−PANI (b).

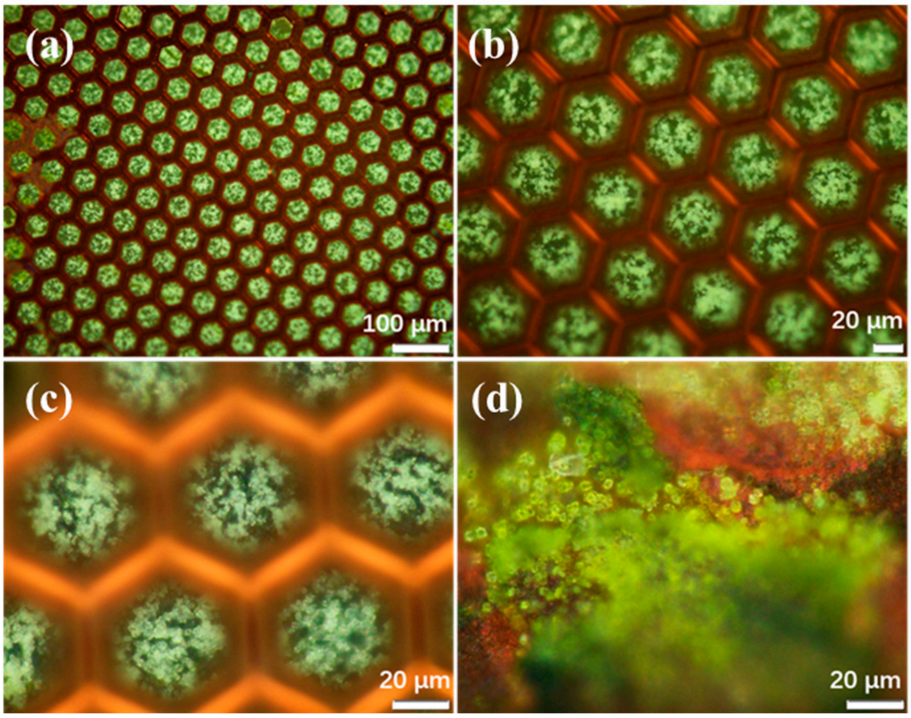

Figure S2. (a−d) Optical microscopy images of F−AN on the copper mesh.

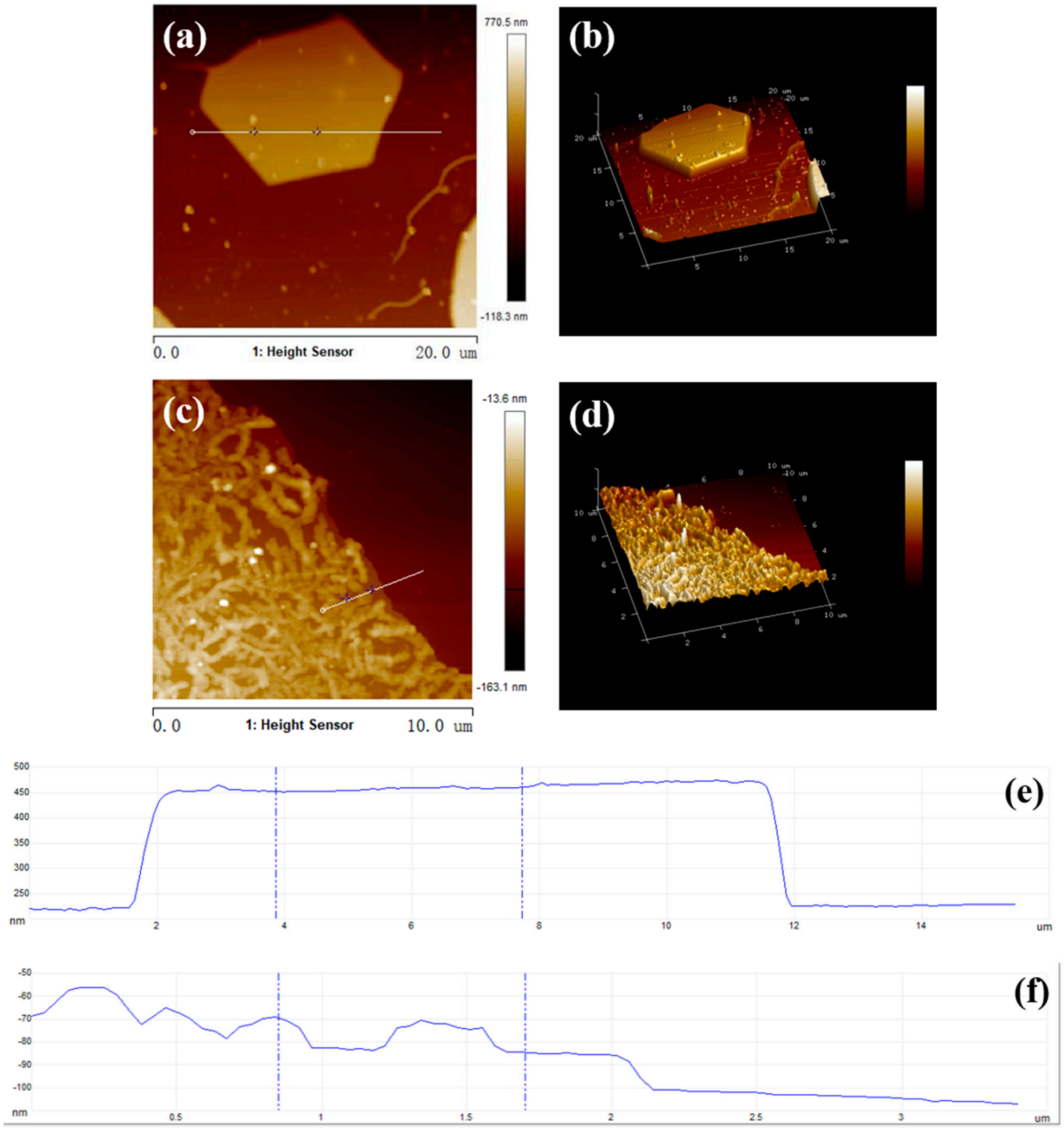

Figure S3. AFM images of F−PANI (a−b) and A−PANI (c−d). The height profiles of the image of F−PANI (e) and A−PANI (f).

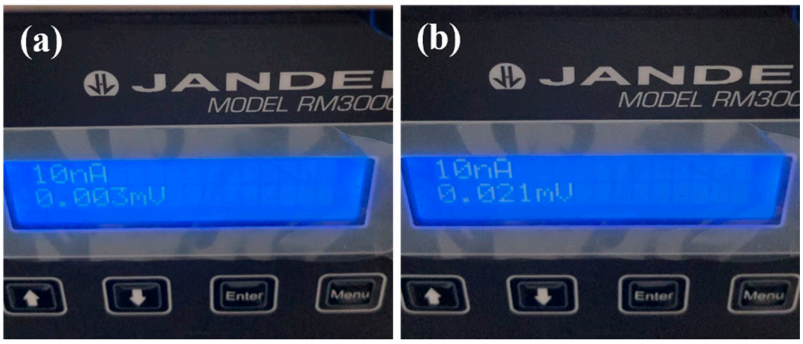

Figure S4. The numerical value of I/V of the F−PANI film (a) and A−PANI film (b).
